# CRP/Neopterin Ratio and Neuropsychiatric Symptoms in Patients with Different Forms of Pneumonia: Results of a Pilot Study

**DOI:** 10.3390/microorganisms12061099

**Published:** 2024-05-29

**Authors:** Katharina Konstanze Lilly Wagner, Daniele Corda, Andreas Steinmayr, Francesco Burkert, Dietmar Fuchs, Johanna Gostner, Stefanie Hofer, Lucia Parrakova, Irina Gasslitter, Günter Weiss, Christian Irsara, Sarah Maier, Andrea Griesmacher, Rosa Bellmann-Weiler, Katharina Kurz

**Affiliations:** 1 Department of Internal Medicine II, Innsbruck Medical University, 6020 Innsbruck, Austria; katharina-wagner2@web.de (K.K.L.W.); daniele.corda@sabes.it (D.C.); andreas.steinmayr@klinikum-wegr.at (A.S.); francesco.burkert@i-med.ac.at (F.B.); guenter.weiss@i-med.ac.at (G.W.); 2 Institute of Biological Chemistry, Biocenter, CCB, Innsbruck Medical University, 6020 Innsbruck, Austria; dietmar.fuchs@i-med.ac.at; 3 Institute of Medical Biochemistry, Biocenter, CCB, Innsbruck Medical University, 6020 Innsbruck, Austria; johanna.gostner@i-med.ac.at (J.G.); steffi.hofer@gmx.at (S.H.); lucia.parrakova@i-med.ac.at (L.P.); 4 Department of Dermatology, Innsbruck Medical University, 6020 Innsbruck, Austria; irina.gasslitter@tirol-kliniken.at; 5 Central Institute for Medical and Chemical Laboratory Diagnostics, University Hospital of Innsbruck, 6020 Innsbruck, Austria; christian.irsara@tirol-kliniken.at (C.I.); andrea.griesmacher@tirol-kliniken.at (A.G.); 6 Institute of Medical Statistics and Informatics, Innsbruck Medical University, 6020 Innsbruck, Austria; sarah.maier@i-med.ac.at

**Keywords:** pneumonia, *influenza*, COVID-19, Neopterin, biomarkers, interferon gamma

## Abstract

Background: Pneumonia is one of the most common infectious diseases, mostly caused by viruses or bacteria. In response to bacteria or viruses which are different but which also are partly overlapping, innate and adaptive immune responses are induced, which can be quantified using the determination of specific biomarkers. Among these, C-reactive protein (CRP) has been established as a marker of innate immune function, whereas Neopterin, which is mainly produced upon stimulation with interferon-gamma, reflects cellular immune activation. Aim: We investigated inflammation markers in patients with microbiologically confirmed viral or bacterial pneumonia, and studied the potential of CRP, Neopterin, and the CRP/Neopterin ratio to distinguish between viral and bacterial pathogenesis. Furthermore, we examined, how often neuropsychiatric symptoms occur in patients suffering from different kinds of pneumonia. Patients and method: A total of 194 patients diagnosed with either coronavirus disease 2019 (COVID-19) (n = 63), bacterial pneumonia (n = 58), *Influenza* infection (n = 10), *Influenza* and a bacterial superinfection (n = 9), and COVID-19 patients with a bacterial superinfection (n = 54) were included in our pilot study. Clinical as well as laboratory parameters were determined shortly after admission. Results: We found significantly higher CRP/Neopterin ratios in patients with bacterial pneumonia (median: 0.34) and lower CRP/Neopterin ratios in patients hospitalized with COVID-19 infection (median: 0.03; *p* < 0.001). Both in men and in women, the CRP/Neopterin ratio was able to distinguish between viral and bacterial pathogens, but also was able to detect bacterial super-infection (BSI) in subjects with initial viral pneumonia (*p* < 0.001). Patients with BSI presented with significantly lower CRP/Neopterin ratios (median 0.08) than patients with bacterial infection only (median 0.34; *p* < 0.001). Interestingly, COVID-19 patients had a decreased physical functioning (as reflected in the ECOG score) and a higher frequency of fatigue (84.1%) and neurological symptoms (54.8%) than patients with pneumonia, due to other underlying pathogens. Patients that reported fatigue during viral and bacterial pneumonia presented with lower CRP concentrations than patients without it. Conclusions: The CRP/Neopterin ratio is useful to differentiate between viral and bacterial pathogenesis. The occurrence of neuropsychiatric symptoms in pneumonia appears to depend on the kind of pathogen causing the infection. Lower CRP concentrations at admission appear to be related to fatigue during acute viral and bacterial infection.

## 1. Introduction

Pneumonia is the most common infectious disease leading to death in industrialized countries. In Europe, hospitalization is required in up to 50% of cases [1]. Pneumonia can be caused by different pathogens, mainly bacteria and viruses, and can often progress to multi-organ disease. Differentiation between viral and bacterial pneumonia is often challenging. However, it is essential for further adequate treatment. Microbiological cultures are helpful to diagnose underlying pathogens. However, in recent years, as results of microbiological cultures are often available only after several days, underlying pathogens are mostly diagnosed by antigen rapid tests or polymerase chain reaction (PCR).

In addition to microbiological tests, the monitoring of the immune response as reflected by inflammation parameters can help to estimate the extent of disease and differentiate between viral and bacterial pathogens. C-reactive protein is one of the acute phase inflammatory proteins. It is induced by interleukin (IL)-6 and IL-1 in the case of infection or inflammation. CRP is particularly elevated during bacterial infections. In cases of viral or fungal infection, CRP levels are usually not increased or are only slightly elevated [2]. Neopterin, on the other hand, is a marker of cellular immune activation, and is mainly produced by monocytes and macrophages after IFN-y stimulation. In contrast to CRP, Neopterin production is mainly increased in viral infections like HIV infection or measles [3]. In the case of bacterial infections, Neopterin formation plays a minor role due to the predominance of the humoral immune response [4,5]. The ratio of CRP/Neopterin has earlier been shown to be useful to differentiate between bacterial or viral pathogens in acute respiratory tract infections [6]. Also, in patients with chronic obstructive pulmonary disease, a higher CRP/Neopterin ratio was found in patients with community-acquired pneumonia (CAP) and COPD compared to patients with acute exacerbations of COPD without pneumonia [7].

CAP is the most common form of pneumonia caused by bacteria. It is defined by developing outside the hospital or within the first 48 h of hospitalization, and is a leading cause of morbidity and mortality worldwide. The most common pathogen leading to CAP is *Streptococcus pneumoniae*, followed by *Haemophilus influenzae*. Atypical pneumonia, on the other hand, can be caused by *Mycoplasma* or *Chlamydia*, and usually goes along with a rather slow onset [4].

*Influenza* infection usually has its peak during the cold season, and annually causes up to 5 million severe infections globally, which can be a burden for public health [8]. *Influenza* A (IAV) is highly susceptible to antigenic variation, and therefore poses a considerable risk of triggering an epidemic or pandemic.

SARS-CoV-2 virus already challenged health care systems worldwide significantly over the years by spreading very quickly and affecting billions of people worldwide. Interestingly, infection by this virus went along with very different courses of disease, varying from asymptomatic to acute respiratory distress syndrome (ARDS) [9]. 

Secondary bacterial superinfection can occur in patients infected with viral infections like SARS-CoV-2 and those with *Influenza* [10,11]. Many mechanisms have been identified for increased bacterial susceptibility after *Influenza* infection, like, e.g., disruption of the barrier function of the alveolar epithelium [12]. SARS-CoV-2, on the other hand, also seems to impair the function of many different cells of the host. It does not only infect alveolar epithelial cells and endothelial cells, but also harms cells of the extracellular matrix and induces the release of various pro-inflammatory cytokines (including IL-1, IL-6, tumor necrosis-factor alpha (TNF-α), and transforming growth factor beta), thereby destroying the “blood gas barrier” [13]. Additionally, SARS-CoV-2 was observed to disrupt the activity of macrophages through pathways like TLR4 and 5, potentially facilitating bacterial attachment and leading to secondary infections [14].

Apart from the abovementioned pathways, viral infection can also induce mucosal cell death, compromising the body’s ability to clear pathogens and promoting bacterial adhesion [15]. Interactions between the virus and host cells can trigger the production of pro-inflammatory markers like TNF-α, which may harm host cells and increase susceptibility to opportunistic bacterial infections [16]. The most common pathogen for secondary bacterial infection is *Staphylococcus aureus*, followed by *Haemophilus influenzae* and *Pseudomonas aeruginosa* [10,12]. These pathogens interfere with the body’s normal functions by impeding immune responses and generating harmful toxins [17].

Therefore, different respiratory pathogens appear to use different mechanisms to invade host cells and also attack host immune cells. And in fact, also, the integrity of the barriers of the airways, and especially the overall “fitness” of the host’s immune system (i.e., the ability of certain immune cells to fight and eliminate pathogens), play a pivotal role in the course of disease in patients with pneumonia. 

Also, the development of certain symptoms might in fact be altered by the interaction of pathogens and the immune system, e.g., the immune system tries to deprive pathogens of essential nutrients like, e.g., tryptophan [18]. Earlier studies showed that inflammation and interferon-gamma mediated biochemical pathways, like Neopterin formation and tryptophan catabolism, are induced in patients with *Influenza* ([19]), patients with acute exacerbated COPD [7], Epstein–Barr virus infection [20], and also pneumonia [21]. In patients with pneumonia, enhanced immune response and tryptophan catabolism were associated with a worse prognosis of patients [21], while other studies also showed that over-activated inflammation and induction of interferon gamma-related biochemical pathways were associated with symptoms/an impaired performance of patients, e.g., in patients with acute COVID-19 infection [22], persistent symptoms after COVID-19 [6,23], and patients with Epstein–Barr virus infection [20]. As higher Neopterin concentrations and enhanced tryptophan catabolism have also been related with symptoms like anemia, fatigue, and depression in earlier studies [24], we also wanted to investigate whether the symptoms of patients are related to inflammatory markers, and especially the CRP/Neopterin ratio, in patients with different kinds of pneumonia. 

In our pilot study, we thus also investigated the frequency of certain common symptoms (fatigue and impaired physical function, depression, sleep disturbance, and neurological symptoms) in patients with different kinds of pneumonia, and retrospectively analyzed whether inflammatory markers were related with those symptoms and were dependent on the involved pathogen. We were especially interested in these symptoms, as many patients after the acute COVID-19 infection also suffer from such symptoms persistently. 

## 2. Patients and Methods

### 2.1. Patient Recruitment and Data Collection

Our study population included 194 patients who were hospitalized at the University Hospital for Internal Medicine II in Innsbruck between 2016 and 2020. Among them, 63 patients had a COVID-19 infection, 58 had a bacterial pneumonia, 10 suffered from *Influenza*, 9 had *Influenza* and an additional bacterial superinfection, and, in addition, 54 COVID-19-infected patients had bacterial superinfections. 

Clinical symptoms, and in particular neuropsychiatric symptoms, were noted at the time of admission. Routine laboratory parameters were determined in all patients, including CRP, complete and differential blood count, and Neopterin. Aliquots of the plasma samples were frozen until the analysis of the relationship between the laboratory parameters and the clinical symptoms. The patients gave informed consent for their blood to be used for scientific research, and the study was approved by the ethical board of the Medical University of Innsbruck. Only individuals older than 18 years were included in the study, and all personal data of patients were pseudonymized through consecutive numbering. The study was approved by the Ethics Committee of the Medical University Innsbruck (EK number: 1167/2020).

### 2.2. Laboratory Examinations

The blood sampling was performed during the first 3 days of their admission to hospital. 

Routine laboratory values were analyzed by the Central Institute for Medical and Chemical Laboratory Diagnostics in Innsbruck, Austria. 

CRP was analyzed using a Cobas 8000 platform (Roche Diagnostics, Rotkreuz, Switzerland).

Neopterin measurements were performed at the Institute of Medical Biochemistry Biocenter of the Medical University of Innsbruck. Neopterin concentrations were measured using an enzyme-linked immunosorbent assay (BRAHMS GmbH, Hennigsdorf, Germany) following the manufacturer’s protocol (sensitivity, 2 nmol/L). 

### 2.3. Statistical Analysis

The statistical analysis was performed with IBM SPSS Statistics, version 28 (IBM Corporation, Armonk, New York, NY, USA). All statistical tests were two-sided, and *p* values < 0.05 were considered to be statistically significant.

Since not all data showed normal distribution in the Shapiro–Wilk test, non-parametric tests were applied. To compare two independent samples, the Mann–Whitney test was used. For more than two independent samples, the Kruskal–Wallis test with pairwise post hoc tests and the Bonferroni correction for multiple testing were used. Differences in ECOG performance status were assessed with the Chi-squared test. Figures were created with IBM SPSS Statistics, version 28 (IBM Corporation, Armonk, New York, NY, USA). 

## 3. Results

### 3.1. Baseline Characteristics and Symptoms of the Study Population

A total of 194 patients admitted to the inpatient ward of the University Hospital of Internal Medicine II between 2016 and 2020 were recruited. In total, 62.4% of the patients (n = 121) were males, and 37.6% were females (n = 73). The age at the time of admission ranged from 19 to 95 years (median: 70). 

The cohort was subcategorized according to the different pathogens causing pneumonia, namely, *SARS-CoV-2* (n = 63; 32.5%), bacteria (n = 58; 29.9%), *Influenza* virus (n = 10; 5.2%), *Influenza* and bacterial superinfection (n = 9; 4.6%), and COVID-19 and superinfection (n = 54; 27.8%). 

Interestingly, especially in patients with COVID-19, the proportion of males predominated (73% vs. 27%). This was also found in COVID-19 plus bacterial superinfection (75.9% vs. 24.1%). Patients with bacterial pneumonia, on the other hand, were mostly women (59% vs. 41%). Thus, gender comparisons were also performed. Men and women did not differ significantly regarding CRP and Neopterin concentrations. However, the CRP/Neopterin ratio was tendentially higher in women in the whole population (*p* = 0.055). 

In terms of origin, among the patients with bacterial pneumonia and *Influenza* pneumonia, nearly all were living in Austria. Among the COVID-19 patients, 24 were from foreign countries (but all were from Europe, and thus were Caucasian).

Smoking status was different in patients with pneumonia. While only seven COVID-19 infected patients were smokers (4.4%), the percentage of smokers in *Influenza* patients (40%) and in patients with bacterial superinfection was higher (10.3%). Out of 58 patients with bacterial pneumonia, 10 were smokers (17.2%).

### 3.2. Laboratory Parameters in Patients with Infection

**Figure 1:**
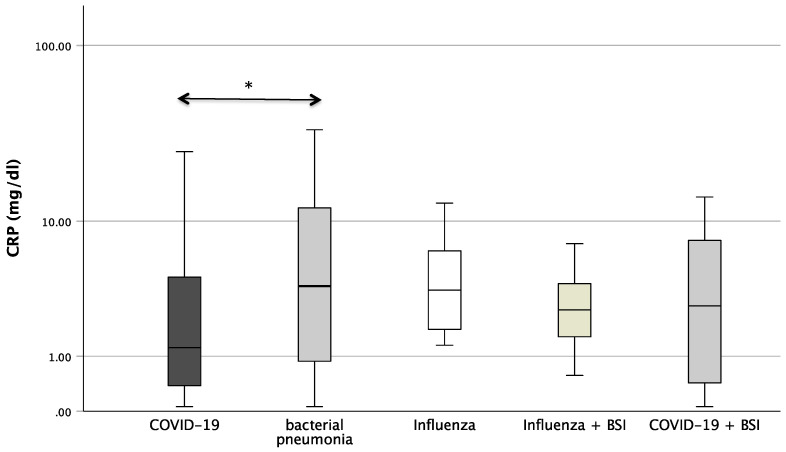

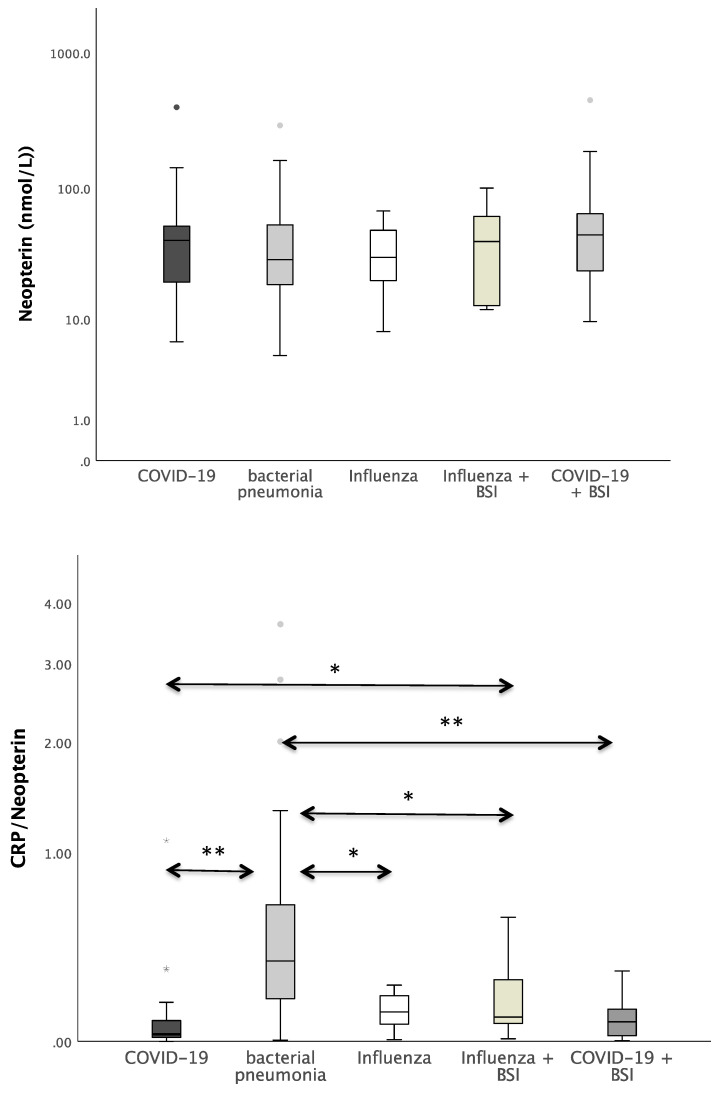
Boxplots of CRP, Neopterin, and CRP/Neopterin in patients with different kinds of pneumonia (* *p*-value < 0.05, ** *p*-value < 0.001). Values that have more than three IQR values from the end of a box are marked with an asterisk (*). Values above 1.5 IQR values but less than 3 IQR values at the end of the box are labelled as outliers (o). The graphs were displayed in different colours to make the different pathogens more visible.

Figure 1 shows the boxplots of the investigated laboratory parameters (CRP, Neopterin, CRP/Neopterin) at admission for the whole cohort of patients with pneumonia divided by the pathogen subgroups. Regarding CRP, a significant difference was found between the pathogens (*p* = 0.034). In the pairwise post hoc tests, COVID-19 patients presented with significantly lower CRP levels than patients with a bacterial infection (*p* = 0.003). In addition, significant differences were also found with regard to CRP/Neopterin (*p* < 0.001). COVID-19 patients had lower CRP/Neopterin ratios compared to bacterial pathogens (*p* < 0.001) and compared to *Influenza* patients with superinfection (*p* = 0.035). Furthermore, patients with bacterial pneumonia showed higher CRP/Neopterin ratios than patients with COVID-19 superinfection (*p* < 0.001) and than patients with *Influenza* (*p* = 0.034) and *Influenza* superinfection (*p* = 0.038), as shown in Figure 1. 

**Figure 2:**
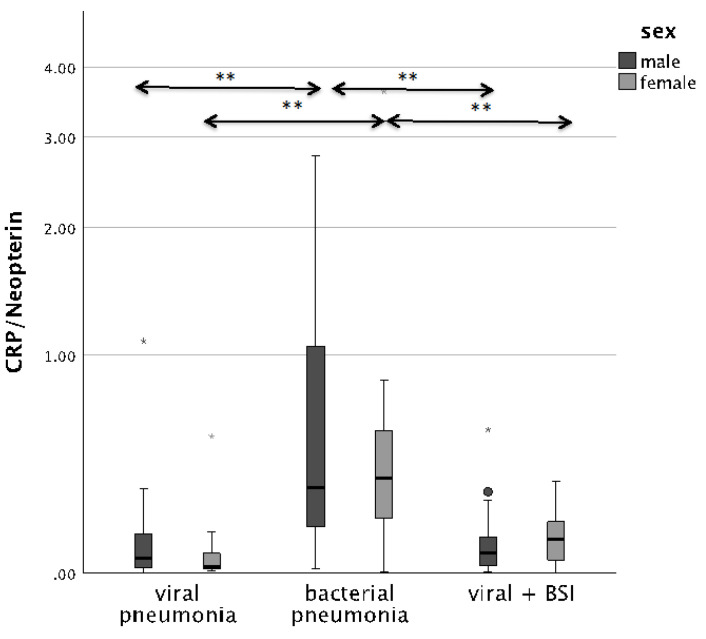
Boxplots comparing the CRP/Neopterin ratio in male patients (left, dark-grey boxplots) and female patients (right, light-grey boxplots) with different pneumonia pathogens (** *p*-value < 0.001). Values that have more than three IQR values from the end of a box are marked with an asterisk (*). Values above 1.5 IQR values but less than 3 IQR values at the end of the box are labelled as outliers (o).

The CRP/Neopterin ratio was able to differentiate between viral and bacterial pathogenesis at admission, as well as superinfections, both in men and in women. Patients with BSI presented with significantly lower CRP/Neopterin ratios than patients with bacterial infection only, as shown in Figure 2 (*p* < 0.001).

### 3.3. Physical Functioning and Clinical Symptoms

As shown in Table 1, there were significant differences regarding the physical functioning of patients. ECOG Performance status > 4 (ECOG 4: completely disabled, incapable of any selfcare, and totally confined to bed or chair; or ECOG 5: consecutive death) was encountered in patients with COVID-19 infection more than twice as often as in other pathogen subgroups. 

Interestingly, we found differences in the frequency of symptoms between the pathogen groups (Table 2). COVID-19 infected patients presented significantly more often with fatigue than *Influenza*-infected patients (84.1% vs. 0%; *p* < 0.001) and significantly more often with neurological symptoms than in *Influenza*-infected patients (54.8% vs. 0%; *p* = 0.003). Patients with bacterial pneumonia likewise suffered from fatigue more often than *Influenza* patients (58.6% vs. 0%; *p* < 0.001). Moreover, male patients suffered more often from fatigue than women during acute infection (74.4% vs. 58.9%; *p* = 0.025). Regarding the age groups, patients older than 65 years had fatigue (78.6% vs. 62.9%; *p* = 0.020) and neurological symptoms (53.6% vs. 36.3%; *p* = 0.020) significantly more often.

### 3.4. Laboratory Parameters and Clinical Symptoms

We also investigated whether patients with/without different symptoms (fatigue, neurological symptoms, depression, and sleep disorder) differed regarding inflammatory parameters. Patients who reported fatigue had unexpectedly lower CRP concentrations at admission than patients without fatigue (see Table 3). Regarding the other observed neuropsychiatric symptoms, no significant differences were found.

As our cohort included more men (especially with COVID-19 infection) and the CRP/Neopterin ratio tended to differ between women and men, we also examined whether there were possible gender differences. In fact, only men with fatigue presented with significantly lower CRP concentrations at admission (*p* = 0.035).

To further clarify whether inflammatory markers differed between patients with/without fatigue depending on pathogens, we analyzed the parameters for the different subgroups (except for *influenza*, as patients did not report fatigue; see Table 4).

**Figure 3:**
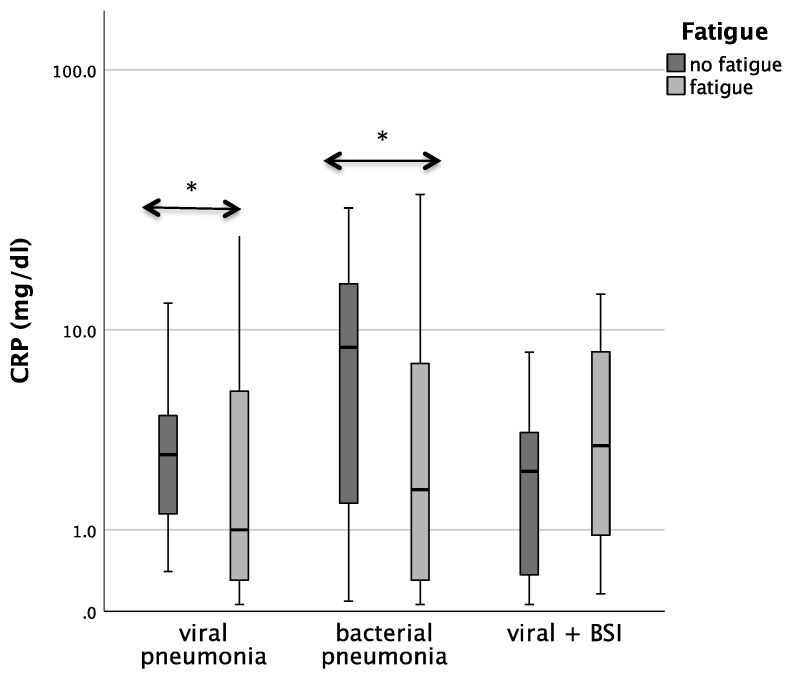

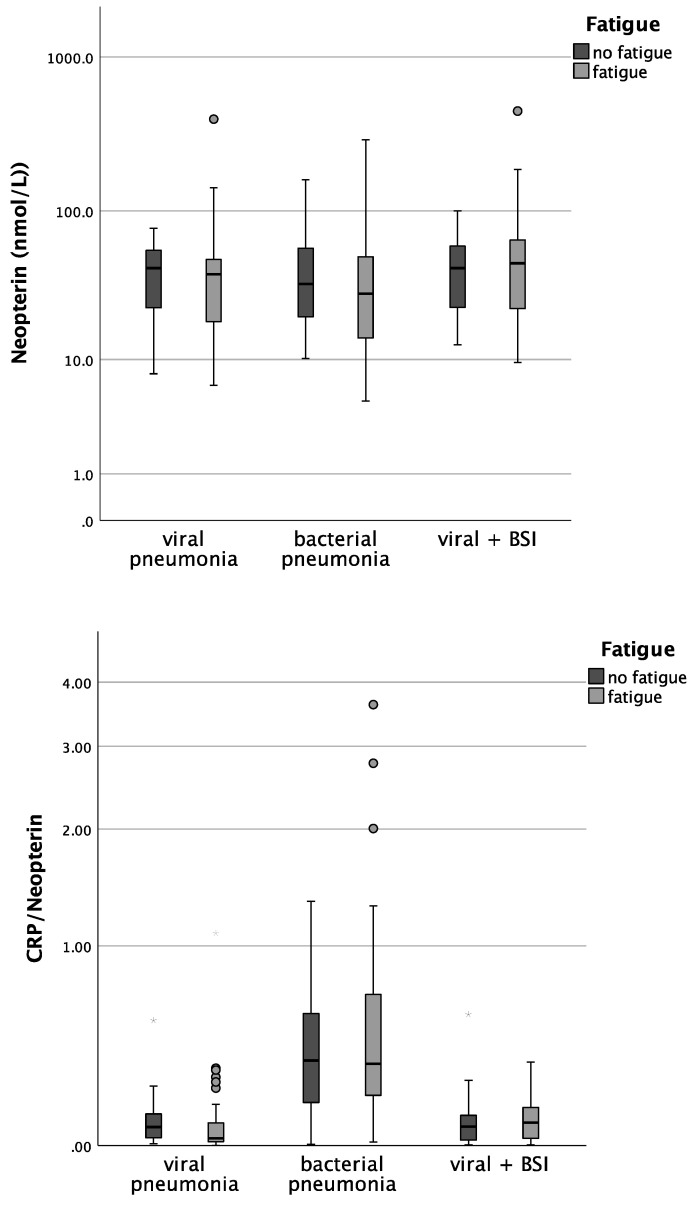
Boxplots comparing CRP, Neopterin, and CRP/Neopterin in patients without fatigue (left, light-grey boxplots) and in patients with fatigue (right, dark-grey boxplots) with different pneumonia pathogens (* *p*-value < 0.05). Values that have more than three IQR values from the end of a box are marked with an asterisk (*). Values above 1.5 IQR values but less than 3 IQR values at the end of the box are labelled as outliers (o).

Patients with bacterial pneumonia and fatigue had significantly lower CRP concentrations than patients without fatigue (median 1.8 vs. 8.5; *p* = 0.029). In patients with COVID-19 pneumonia. CRP/Neopterin was tendentially lower in patients with fatigue (*p* = 0.071, see Figure 3). 

Also, the analysis in the whole cohort confirmed that CRP concentrations in both viral as well as bacterial pneumonia were lower in patients with fatigue during acute infection (*p* = 0.029 for bacterial pneumonia, *p* = 0.048 for viral pneumonia). 

Interestingly, we found differences in the frequency of symptoms between the pathogen groups (Table 5). COVID-19-infected patients presented significantly more often with fatigue than *Influenza*-infected patients (84.1% vs. 0%; *p* < 0.001) and presented significantly more often with neurological symptoms than *Influenza*-infected patients (54.8% vs. 0%; *p* = 0.003). Patients with bacterial pneumonia likewise suffered from fatigue more often than *Influenza* patients (58.6% vs. 0%; *p* < 0.001). Moreover, male patients suffered more often from fatigue than women during acute infection (74.4%vs. 58.9%; *p* = 0.025). Regarding the age groups, patients older than 65 years had significantly fatigue (78.6% vs. 62.9%; *p* = 0.020) and neurological symptoms (53.6% vs. 36.3%; *p* = 0.020) more often.

As our cohort included more men (especially with COVID-19 infection) and the CRP/Neopterin ratio tended to differ between women and men, we also examined whether there were possible gender differences. In fact, only men with fatigue presented with significantly lower CRP concentrations at admission (*p* = 0.035).

To further clarify whether inflammatory markers differed between patients with/without fatigue depending on pathogens, we analyzed the parameters for the different subgroups (except for *influenza*, as patients did not report fatigue; see Table 6).

Patients with bacterial pneumonia and fatigue had significantly lower CRP concentrations than patients without fatigue (Table 6, median 1.8 vs. 8.5; *p* = 0.029). In patients with COVID-19, pneumonia CRP/Neopterin was tendentially lower in patients with fatigue (*p* = 0.071). 

Also, the analysis in the whole cohort confirmed that CRP concentrations in both viral as well as bacterial pneumonia were lower in patients with fatigue during acute infection (*p* = 0.029 for bacterial pneumonia, *p* = 0.048 for viral pneumonia). 

## 4. Discussion

Viral and bacterial respiratory diseases have been a major threat for the global health system in recent decades, even before COVID-19. Within this pilot study, we wanted to investigate the potential of the CRP/Neopterin ratio to distinguish between viral and bacterial pneumonia. 

In patients admitted to hospital with pneumonia, a routine lab test was performed to guide therapy and choose whether antibiotics were necessary or not. In cases of bacterial infection, CRP and leukocytes are usually increased, whereas increased lymphocyte counts and Neopterin levels are observed in viral infections [25]. This was also the case in our study population. CRP levels were highest in bacterial pneumonia, followed by *Influenza*. Moreover, CRP levels were higher in patients older than 65 years. The CRP/Neopterin ratio differed significantly between patients with viral and bacterial pneumonia in our cohort, and was lowest in patients hospitalized with COVID-19 infection and highest in patients with bacterial pneumonia. The ratio was, as expected, age-dependent, being higher in the >65 years age group than in the <65 years group. This is probably, amongst other reasons, due to the higher release of cytokines in elderly people, as well as due to the decrease in innate and adaptive immune responses [26].

As in our study population, gender distribution was different depending on the underlying pathogens. We also performed additional analyses to examine whether gender-related differences might play a role. COVID-19 patients were mostly men (73%), while patients with bacterial pneumonia were mostly women (59%). This finding is well in line with clinical observations; in fact, men have a higher risk of a severe course of COVID-19 and a 61% higher mortality than women [27].

Both in men and in women, the CRP/Neopterin ratio was useful to distinguish between bacterial and viral infections in patients with pneumonia, and was also suited better to differentiate bacterial superinfection compared to CRP alone. To our knowledge, our pilot study shows, for the first time, that the CRP/Neopterin ratio can also distinguish between bacterial superinfection and viral or bacterial pneumonia.

Due to the increase in antibiotic resistance worldwide, it is essential to have biomarkers to guide whether antibiotic treatment is necessary or not, and the CRP/Neopterin ratio might in fact be a very useful biomarker to assist this decision in the early phase of infection. 

In general, there are also only a few studies comparing all three groups of pathogens in regard to the abovementioned inflammatory parameters, and also the frequency of extrapulmonary symptoms has—at least to our knowledge—never been compared for different pathogens.

Data of this pilot study confirm our clinical observation, namely, that COVID-19 patients had significantly more neuropsychiatric symptoms such as fatigue or neurological symptoms in comparison to other pneumonia patients, alongside had a worse physical functioning. Interestingly, there were also differences regarding inflammatory marker CRP between patients with and without fatigue, indicating that inflammatory processes could be involved in the development of symptoms in patients with acute infection. However, rather unexpectedly, we found lower CRP concentrations at admission in patients reporting fatigue. In fact, we would rather have expected the opposite: namely, higher inflammatory markers in patients with fatigue. Earlier data had shown higher CRP and Interleukin-6 levels in 31 patients with sleep disturbance during acute COVID-19 infection, and higher CRP concentrations in patients with persistent fatigue 60 days after acute COVID-19 infection [6]. However, in that earlier study, only COVID-19 patients were included, while in this study we compared patients with different kinds of pneumonia in a cohort comprising significantly more patients. Another explanation for our finding could be that those patients, who had very low energy levels (and thus probably impaired mitochondrial function of many cells in the body, also including immune and liver cells) also had reduced capacities to produce CRP and fight the pathogens adequately. 

Irrespective of the cause, longitudinal studies investigating the dynamics of inflammatory parameters, and especially the CRP/Neopterin ratio during the course of acute infections and reconvalescence depending on underlying pathogens, would certainly be useful to monitor the immune–host interaction even more precisely, especially in patients with COVID-19 infection, as these patients often suffer from persistent symptoms for weeks or even years—a syndrome also called Long COVID [28]. Following their recovery from the initial illness, approximately 10 to 20% of COVID-19-infected patients, as estimated by the World Health Organization (WHO), are anticipated to encounter mid- to long-term effects like fatigue/impaired physical function, neurological symptoms, sleep disorder, and depression [29,30,31]. 

An increased incidence of cognitive disabilities, as well as depressed mood and anxiety, has been reported during acute infection and thereafter [32]. However, other pathogens can also induce neuropsychiatric symptoms in pneumonia patients [33]. Neurological complications have been linked to both *Influenza* type A and *Influenza* type B. Additionally, individuals diagnosed with *Influenza* also appear to be at a higher risk of developing depression later in life [34]. 

The frequency of depression during acute infection did not differ significantly between pathogens in our population. On the other hand, COVID-19 patients were affected by fatigue significantly more often, especially in comparison to *Influenza* patients. Also, the physical functioning of patients with COVID-19 was more strongly impaired compared to infection with other respiratory pathogens, which fits well with our clinical observations. Also, neurological symptoms, such as headaches and smell or taste disorders, were observed significantly more often in COVID-19 patients compared to *Influenza*-infected patients. Several studies have proposed/shown that *SARS-CoV-2* also affects the nervous system [31,35,36] and might affect brain function by inducing neuroinflammatory cascades. 

In a comparison of mice with mild respiratory infection with H1N1 *Influenza* or COVID-19, a similar pathology was observed in the hippocampus. For example, 7 days after infection, in both groups, various neuro-inflammatory cytokines were still elevated in the cerebrospinal fluid, as well as white matter-selective microglial/macrophage reactivity in the subcortical and hippocampal white matter. However, in contrast to COVID-19, the *Influenza*-infected mice showed no lasting effects on subcortical white matter integrity [30]. These findings in mice might also explain why patients suffering from Long COVID might suffer from ongoing deficits of memory, focus, and concentration. 

There are limitations of our pilot study which have to be taken into account. The relatively small number of patients, especially *Influenza* and *Influenza* patients, with a superinfection compared to COVID-19 patients, and the retrospective study design, may limit our general conclusions. Furthermore, repeated measurements of inflammation parameter concentrations and CRP/Neopterin ratio, as well as an assessment of the neuropsychiatric dynamics of the patients, are unfortunately missing. In future studies, it might also be useful to explore the symptoms via standardized questionnaires so that they can be better quantified. Another limitation of our pilot study is certainly that many multimorbid patients were included, and that the study population was rather heterogeneous, which can pose a challenge for comparison and general conclusions.

On the other hand, our study showed interesting new data which merit further investigation in larger longitudinal studies with more homogenous patient populations.

## Figures and Tables

**Table 1:** Number and percentage of patients in the ECOG score categories at admission, divided by pathogens. Significant differences were found between different kinds of pneumonia.

	COVID-19 n (%)	Bacterial Pneumonia n (%)	*Influenza* n (%)	*Influenza* + Other n (%)	COVID-19 + Other n (%)
ECOG (n)					
0 n = 9	0 (0)	0 (0)	0 (0)	0 (0)	9 (16.7)
1 n = 12	9 (14.3)	0 (0)	0 (0)	0 (0)	3 (5.6)
2 n = 74	23 (36.5)	29 (50.0)	4 (44.4)	3 (30.0)	15 (27.8)
3 n = 55	11 (17.5)	23 (39.7)	5 (55.6)	6 (60.0)	10 (18.5)
4 n = 36	17 (27.0)	4 (6.9)	0 (0)	1 (10.0)	14 (25.9)
5 n = 8	3 (4.8)	2 (3.4)	0 (0)	0 (0)	3 (3.6)
Total n = 194	63 (100)	58 (100)	9 (100)	10 (100)	54 (100)
*p*-value	<0.001				

**Table 2:** Comparison between the pathogen groups regarding their clinical symptoms at admission. % of total (n = 194) patients. Significant differences were found regarding neurological symptoms and fatigue.

	COVID-19 n (%)	Bacterial pneumonia n (%)	*Influenza* n (%)	*Influenza* + Other n (%)	COVID-19 + Other n (%)	*p*-Value
Neurological symptoms (n = 82)	34 (54.8)	19 (32.8)	0 (0)	2 (20)	27 (50)	**0.003**
Fatigue (n = 133)	53 (84.1)	34 (58.6)	0 (0)	2 (20)	44 (81.5)	**<0.001**
Depression (n = 12)	4 (6.3)	2 (3.4)	0 (0)	0 (0)	6 (11.1)	n.s.
Sleep disorder (n = 32)	12 (19)	8 (13.8)	0 (0)	0 (0)	12 (22.2)	n.s.
Total n = 194	63 (100)	58 (100)	9 (100)	10 (100)	54 (100)	

**Table 3:** Median concentrations and interquartile range of the investigated laboratory parameters at admission, divided by neuropsychiatric symptoms. CRP differed significantly between patients with fatigue.

		CRP (n = 194)	Neopterin (n = 194)	CRP/Neopterin (n = 194)
MEDIAN (IQR)				
Fatigue	no fatigue (n = 61)	3.2 (1.3–8.0)	37.8 (20.2–57.2)	0.09 (0.03–0.34)
	Fatigue (n = 133)	1.8 (0.3–6.5)	39.1 (19.2–53.6)	0.09 (0.02–0.25)
Neurological symptoms	no neurol. symptoms (n = 112)	2.1 (0.5–6.5)	39.4 (20.0–55.3)	0.09 (0.03–0.30)
	neurological symptoms (n = 82)	2.4 (0.6–8.2)	38.6 (19.5–55.2)	0.08 (0.02–0.20)
Depression	no depression (n = 182)	2.3 (0.5–7.0)	39.4 (20.0–55.9)	0.09 (0.02–0.26)
	depression (n = 12)	2.3 (0.40–7.8)	31.9 (16.0–47.6)	0.06 (0.01–0.22)
Sleep disorder	no sleep disorder (n = 162)	2.4 (0.5–6.9)	38.0 (19.9–53.4)	0.09 (0.03–0.26)
	sleep disorder (n = 32)	1.9 (2.9–11.0)	44.7 (20.8–74.0)	0.07 (0.02–0.22)

**Table 4:** Median concentrations and interquartile range of the investigated laboratory parameters at admission, divided by fatigue. CRP differed significantly in patients with bacterial pneumonia. Mann–Whitney-U tests were performed.

		CRP	Neopterin	CRP/Neopterin
		(n = 194)	(n = 194)	(n = 194)
MEDIAN (IQR)				
COVID-19	no fatigue	2 (0.5–4.2)	53.4 (33.3–57.5)	0.05 (0.02–0.08)
	fatigue	1 (0.3–5.8)	38.2 (18.2–48.2)	0.03 (0.014–0.08)
Bacterial pneumonia	no fatigue	**8.5** (1.5–15.5)	32.9 (19.7–59.1)	0.34 (0.15–0.59)
	fatigue	**1.8** (0.3–7.5)	28.4 (14.0–51.6)	0.33 (0.18–0.72)
COVID-19 BSI	no fatigue	1.4 (0.2–4.4)	42.5 (21.4–62.4)	0.02 (0.005–0.07)
	fatigue	3.1 (0.9–8.4)	45.2 (22.8–68.8)	0.08 (0.03–0.14)

**Table 5:** Comparison between the pathogen groups regarding their clinical symptoms at admission. % of total (n = 194) patients. Significant differences were found regarding neurological symptoms and fatigue.

	COVID-19 + Other n (%)	*Influenza* + Other n (%)	*Influenza* n (%)	Bacterial Pneumonia n (%)	COVID-19 n (%)	*p*-Value
Neurological symptoms (n = 82)	27 (50)	2 (20)	0 (0)	19 (32.8)	34 (54.8)	**0.003**
Fatigue (n = 133)	44 (81.5)	2 (20)	0 (0)	34 (58.6)	53 (84.1)	**<0.001**
Depression (n = 12)	6 (11.1)	0 (0)	0 (0)	2 (3.4)	4 (6.3)	n.s.
Sleep disorder (n = 32)	12 (22.2)	0 (0)	0 (0)	8 (13.8)	12 (19)	n.s.
Total n = 194	54 (100)	10 (100)	9 (100)	58 (100)	63 (100)	

**Table 6:** Median concentrations and interquartile range of the investigated laboratory parameters at admission, divided by fatigue. CRP differed significantly in patients with bacterial pneumonia. Mann–Whitney-U tests were performed.

		CRP/Neopterin	Neopterin	CRP
		(n = 194)	(n = 194)	(n = 194)
MEDIAN (IQR)				
COVID-19	no fatigue	0.05 (0.02–0.08)	53.4 (33.3–57.5)	2.0 (0.5–4.2)
	fatigue	0.03 (0.014–0.08)	38.2 (18.2–48.2)	1.0 (0.3–5.8)
	*p*-value	0.467	0.071	0.455
bacterial pneumonia	no fatigue	0.34 (0.15–0.59)	32.9 (19.7–59.1)	**8.5** (1.5–15.5)
	fatigue	0.33 (0.18–0.72)	28.4 (14.0–51.6)	**1.8** (0.3–7.5)
	*p*-value	0.683	0.439	**0.029**
COVID-19 BSI	no fatigue	0.02 (0.005–0.07)	42.5 (21.4–62.4)	1.4 (0.2–4.4)
	fatigue	0.08 (0.03–0.14)	45.2 (22.8–68.8)	3.1 (0.9–8.4)
	*p*-value	**0.023**	0.725	0.099

## Data Availability

The raw data supporting the conclusions of this article will be made available by the authors on request.
